# Burden, clinical characteristics, and management patterns of prostate cancer in Nigeria: a systematic review and meta-analysis

**DOI:** 10.3389/fonc.2026.1780152

**Published:** 2026-03-30

**Authors:** Wusa Makena, Monday Nwankwo, Aisha Idris, John Tabakwot Ayuba, Victor Archibong, Alex Ogbe, Ahumuza Ronald, Amaka Doris Emelonye, Abdurrasheed Ola Muhammed, Barka Ishaku, Kolawole Bolaji Philip

**Affiliations:** 1 Department of Human Anatomy, Faculty of Biomedical Sciences, Kampala International University, Bushenyi, Uganda; 2 Department of Human Anatomy, Faculty of Basic medical science, Federal University Lafia, Lafia, Nigeria; 3 Department of Human Physiology, Faculty of Basic Medical Sciences, College of Medicine, Kaduna State University, Kaduna, Nigeria; 4 Department of Human Anatomy, Mbarara University of Science and Technology, Mbarara, Uganda; 5 Department of Human Anatomy, School of Medicine and Pharmacy, College of Medicine and Health Sciences, Butare, Huye, Rwanda; 6 Department of Nursing, School of Nursing, College of Medicine, Kampala International University, Bushenyi, Uganda; 7 Department of Human Anatomy, Faculty of Basic Medical Sciences, College of Medicine, University of Maiduguri, Maiduguri, Nigeria

**Keywords:** management, meta-analysis, Nigeria, prostate cancer, public health, risk factors, systematic review

## Abstract

**Background:**

Prostate cancer is one of the most common types of cancer morbidity and mortality among Nigerian men, but there are still limited comprehensive epidemiological data. Understanding the hospital-based patterns of prostate cancer burden, risk factor distributions, and treatment modalities is paramount in supporting policy, clinical practice, and resource allocation in the healthcare system of Nigeria.

**Purpose:**

The aim of the systematic review and meta-analysis was to summarize information regarding the burden, clinical features, and management patterns of prostate cancer in Nigeria.

**Methods:**

A systematic review and meta-analysis conducted in PRISMA 2020 were implemented. PubMed, Scopus, Web of Science, and Google Scholar were searched since January 2000. Research studies that had reported prostate cancer percentage, risk, or treatment patterns among Nigerian men were eligible.

**Results:**

32 studies (19,050 participants) were included, mainly retrospective (44.1) and cross-sectional (41.2), mostly hospital-based (82.4) and were done in Southwest and south-south Nigeria. In 70.6% of the cases, the diagnosis was confirmed using histology. The combined percentage of prostate cancer was 16.4 in hospital-based researches (95% CI: 8.6%29.2; I 2 = 99.3) and 14.0 in population-based research (95% CI: 4.1 - 40.0%; I^2^ = 98.0), with broad prediction intervals. Greater percentages were found in tertiary compared to primary care, in single- compared to multi-centre studies and in populations with an average age over 60 years (p<0.05). The regional estimates were between 5.1 and 33.0. Included in the common risk factors were older age (26.8%), family history (25%), and diet (12.5%). The most widespread therapy (36 percent) was hormonal therapy.

**Conclusions:**

Prostate cancer is a significant problem in healthcare facilities of Nigeria, and radical heterogeneity along with methodological constraints make it impossible to make conclusive estimates regarding prevalence. Existing health disparities and insufficient access to curative therapies are also distinctive to the areas. Cancer control policies in Nigeria are in urgent need of population-based cancer registries, multicentre cohort studies, and implementation research to inform evidence-based cancer control policies.

**Systematic review registration:**

https://www.crd.york.ac.uk/prospero/, identifier CRD420261325315.

## 1 Introduction

Prostate cancer ranks among the major causes of cancer morbidity and mortality in men around the world and most especially in sub-Saharan Africa (1, 2). Prostate cancer is a major public health issue in Nigeria, the most populous country in Africa, but there is still limited epidemiological data on this disease (3). The awareness of the burden, clinical features, and trends in managing prostate cancer in Nigeria is needed to support evidence-based policy, clinical practice optimization, and effective healthcare resource allocation (4).

Prostate cancer aetiology entails both the non-modifiable and modifiable risk factors. The non-modifiable risk factors are age above 50 years, advancing age increases the incidence exponentially, the African ancestry, which is a risk factor than other ethnic groups, and family history of prostate cancer (5, 6). Lifestyle and dietary habits have been identified among the modifiable risk factors which can include excessive intake of red meat and saturated fats, obesity, lack of physical activity, and possibly alcohol consumption, and smoking but the evidence is not consistent (7). There are also studies that have linked comorbid conditions like hypertension and diabetes to the risk of prostate cancer (8).

Prostate cancer in Nigeria presents a lot of management problems. Radical prostatectomy, external beam radiotherapy, brachytherapy, and state of the art systemic therapies are treatments that are usually inaccessible and unaffordable to most of the patients (9–11). Therefore, there are numerous patients that receive advanced disease and depend more on androgen deprivation therapy that is more accessible but it provides only palliative advantage in metastatic cases (12). These issues are further aggravated by limited radiotherapy facilities, excessive out-of-pocket expenditures, and insufficient health insurance coverage as well as the geographical access of the special cancer centres (13, 14).

Although prostate cancer has been established as a significant burden in Nigeria, the available research is mainly hospital-based, heterogenous in methodology and gives different estimates of prevalence (15). Due to the heterogeneity of the studies designs, populations, diagnostic criteria, and reporting standards, it is hard to come up with definitive estimates of disease burden and any agreeable trends of risk factors and management practices (16). The scope of previous reviews has been small, or they have not used rigorous systematic review and meta-analytic methods (17).

To address these gaps, we conducted a PRISMA 2020-compliant systematic review and meta-analysis of the existing literature on prostate cancer in Nigeria. The objectives of this study were to: (I) synthesize available evidence on the hospital-based and population-based proportion of prostate cancer among men attending healthcare facilities in Nigeria; (II) describe the distribution of risk factors among prostate cancer cases; (III) characterize current management patterns and treatment modalities; and (IV) identify gaps in the evidence base to guide future research priorities.

## 2 Methods

### 2.1 Study design and protocol registration

This systematic review and meta-analysis were conducted in accordance with the Preferred Reporting Items for Systematic Reviews and Meta-Analyses (PRISMA) 2020 guidelines (18). The protocol was prospectively registered with PROSPERO (CRD420261325315) prior to data extraction to ensure transparency and minimize reporting bias. The review adhered to established methodological standards for systematic reviews of observational studies (19).

### 2.2 Eligibility criteria

Studies were included if they met the following criteria:

Population: Adult men (≥18 years) in Nigeria

Exposure/Condition: Prostate cancer (confirmed by histology, imaging, PSA, or clinical diagnosis)

Outcomes: At least one of the following: (i) prevalence or proportion of prostate cancer; (ii) risk factor associations or distributions; (iii) management or treatment patterns

Study designs: Cross-sectional, cohort (prospective or retrospective), or interventional studies

**Figure 1:**
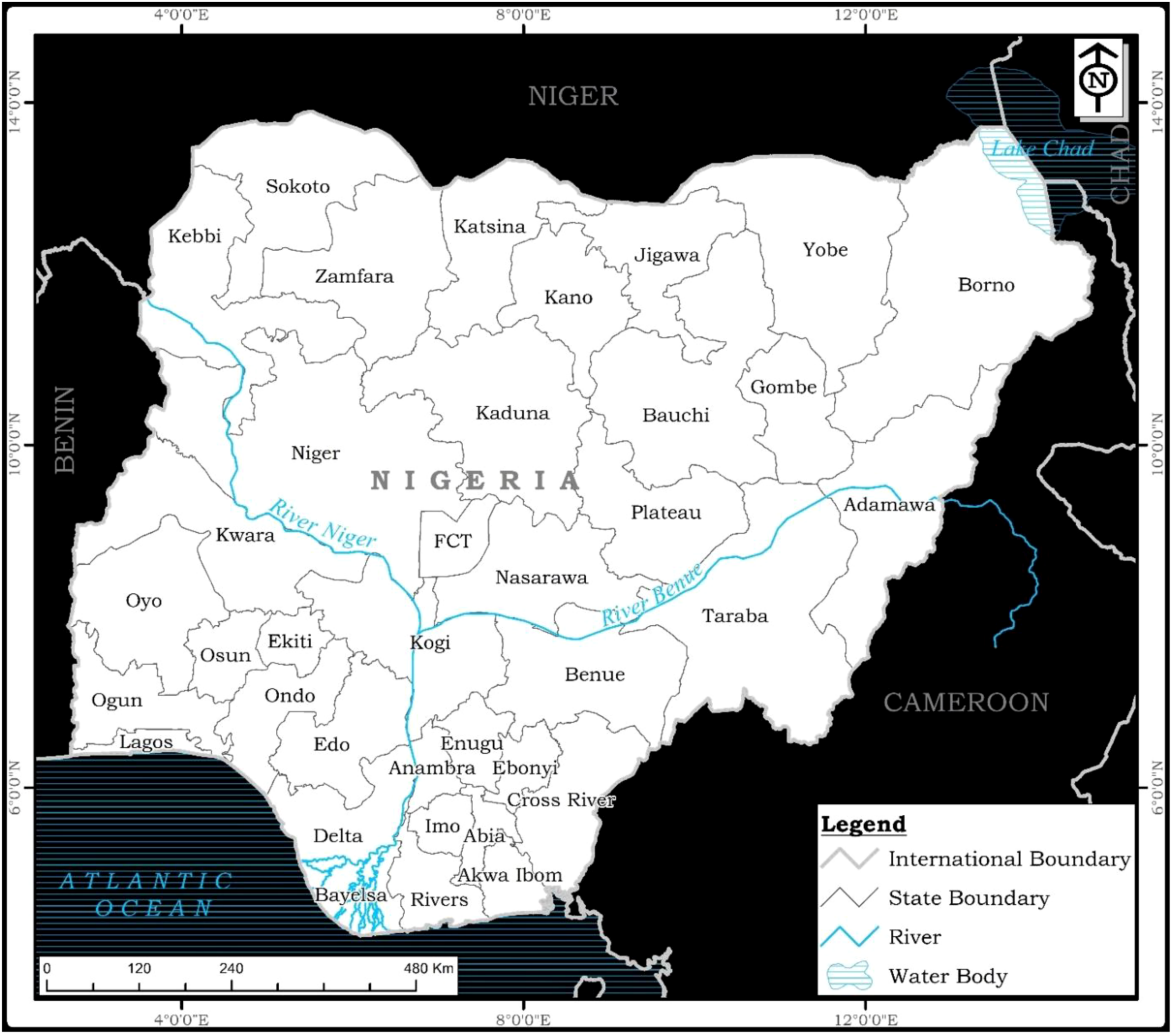
Nigeria. Source: Modified from https://www.openstreetmap.org/#map=6/9.117/8.674.

Setting: Hospital-based, community-based, or population-based studies conducted in Nigeria (**Figure 1**).

### 2.3 Exclusion criteria

Studies were excluded if they: (i) included non-Nigerian participants or participants aged <18 years; (ii) did not report prevalence, risk factor associations, or management data; (iii) were case reports, case series with <10 participants, editorials, commentaries, or reviews without original data; or (iv) had insufficient data for extraction despite attempts to contact authors.

### 2.4 Information sources and search strategy

A comprehensive literature search was conducted across four electronic databases: PubMed/MEDLINE, Scopus, Web of Science, and Google Scholar. The search covered all records from database inception through September 25, 2025. The search strategy combined Medical Subject Headings (MeSH) terms and free-text keywords related to prostate cancer, prevalence, risk factors, management, and Nigeria. The full search strategy for PubMed was as follows: (“prostate cancer” OR “prostatic neoplasm” OR “prostate carcinoma” OR “adenocarcinoma of prostate”) AND (“Nigeria” OR “Nigerian”) AND (“prevalence” OR “incidence” OR “epidemiology” OR “burden” OR “risk factors” OR “management” OR “treatment” OR “therapy”). Similar search strategies were adapted for other databases. No language or publication date restrictions were applied. Reference lists of included studies and relevant review articles were manually screened to identify additional eligible studies.

### 2.5 Study selection

All retrieved records were imported into Rayyan reference management software, and duplicate records were removed prior to the screening process. Duplicate records were automatically and manually removed. Two independent reviewers (WM and MN) screened titles and abstracts against eligibility criteria. Full-text articles of potentially eligible studies were retrieved and independently assessed by the same reviewers. Disagreements were resolved through discussion or consultation with a third reviewer (AI). Reasons for exclusion at the full-text stage were documented.

### 2.6 Data extraction

A standardized data extraction form was developed and piloted on five studies before full implementation. Two reviewers (WM and JTA) independently extracted data from included studies. The information extracted contained the study identification (first author, year of publication, journal, and country or region within the country of Nigeria); the study design and setting (study design, setting (community-based, hospital-based, or population-based study), and sampling method); the population features (sample size, age (mean or median with range), inclusion and exclusion criteria, and the urban or rural location); and condition-specific data (prostate cancer diagnostic criteria; clinical assessment, PSA, and digital rectal examination, histology, and imaging). Prevalence data were also pulled out, including the numerator (number of cases), denominator (number of total sample size), the type of prevalence (point or period), and the confidence intervals. Besides, the data about risk factors were gathered. The data about the management were pulled out, such as the nature of intervention, comparator, outcome measures and follow-up time. Data that was needed to assess quality and risk of bias, also data about funding sources and conflict of interest were recorded. Records were cross-linked to eliminate duplication when several publications used similar population to conduct their study. Any discrepancies in data extraction were resolved through discussion between the reviewers and by referring back to the original publications.

### 2.7 Risk of bias assessment

Risk of bias was assessed independently by two reviewers (WM and VA) using tools study design-appropriate appraisal tools. In the prevalence studies, Joanna Briggs Institute (JBI) Critical Appraisal Checklist of Studies Reporting Prevalence Data was used (20). This tool measures 9 methodological areas such as the suitability of the sample frame, sampling method, sufficient sample size, description of study participants and setting, sufficient data coverage, suitability of condition identification measures, reliability and standardization of measurement, adequacy of statistical analysis and adequacy of response rates. Any differences among reviewers were solved due to consensus or in exceptional cases, the third reviewer was consulted. Risk of bias assessments were summarized in terms of traffic-lights plots and total summary statistics in terms of the percentage of the studies that were rated to be at low, unclear, or high risk of bias in each of the domains. Disagreements were resolved by consensus or third-party adjudication (AO).

### 2.8 Data synthesis and statistical analysis

#### 2.8.1 Meta-analysis of proportions

For Proportion outcomes, we conducted random-effects meta-analysis using the Restricted Maximum Likelihood (REML) method to estimate between-study variance (τ²). REML was selected because it provides less biased and more reliable variance estimates than traditional moment-based approaches, particularly in the presence of substantial heterogeneity and a moderate number of studies (21, 22). This method enhances the accuracy and stability of pooled estimates while appropriately accounting for between-study variability. Proportions were transformed using the logit transformation to stabilize variance and ensure that confidence intervals remained within the 0–1 range (23). Pooled proportions were back-transformed for presentation. Given the expected heterogeneity in hospital-based observational studies with varying populations, diagnostic criteria, and healthcare settings, we justified pooling by conducting extensive subgroup and sensitivity analyses to explore sources of heterogeneity and assess the robustness of findings (24).

Heterogeneity was quantified using Cochran’s Q test (with p < 0.10 indicating significant heterogeneity), the I² statistic (with values of 25%, 50%, and 75% representing low, moderate, and high heterogeneity, respectively) (25), Tau² to estimate between-study variance, and 95% prediction intervals to determine the range of true effects expected in future studies (26).

#### 2.8.2 Subgroup and meta-regression analyses

For risk factor and management outcomes, subgroup analyses were conducted by stratifying studies according to geographic region (North-Central, Northeast, Northwest, Southeast, South-South, and Southwest), study setting (hospital-based versus population-based), level of care (primary versus tertiary), number of centers (single-center versus multi-center), sample size (≤100 versus >100), mean age of participants (≤60 years versus >60 years), and data collection period (1995–2015 versus 2016–2026). In addition, random-effects meta-regression was performed to examine the associations between study-level covariates, including mean age and year of data collection, and the log odds of prostate cancer proportion. The proportion of between-study variance explained by the covariates (R² analog) was also calculated (27).

#### 2.8.3 Sensitivity analysis and publication bias

Sensitivity analyses were conducted by sequentially removing one study at a time (leave-one-out analysis) to assess the influence of individual studies on pooled estimates. Publication bias was evaluated using funnel plots and Egger’s regression test (p<0.10 indicating potential bias) (28). Trim-and-fill analysis was performed to estimate the number of potentially missing studies and adjust pooled estimates accordingly (29). All statistical analyses were performed using Comprehensive Meta-Analysis (CMA) software version 4.0 (Biostat, Englewood, NJ, USA). Statistical significance was set at p<0.05 (two-tailed) except where otherwise specified.

### 2.9 Certainty of evidence assessment

The certainty of evidence for each outcome was assessed using the Grading of Recommendations Assessment, Development and Evaluation (GRADE) approach (30). Evidence from observational studies started at low certainty and was rated down for risk of bias, inconsistency (heterogeneity), indirectness, imprecision, and publication bias. Evidence could be rated up for large magnitude of effect, dose-response gradient, or if all plausible confounding would reduce the observed effect. Final certainty ratings were classified as high, moderate, low, or very low.

## 3 Results

### 3.1 Study selection

**Figure 2:**
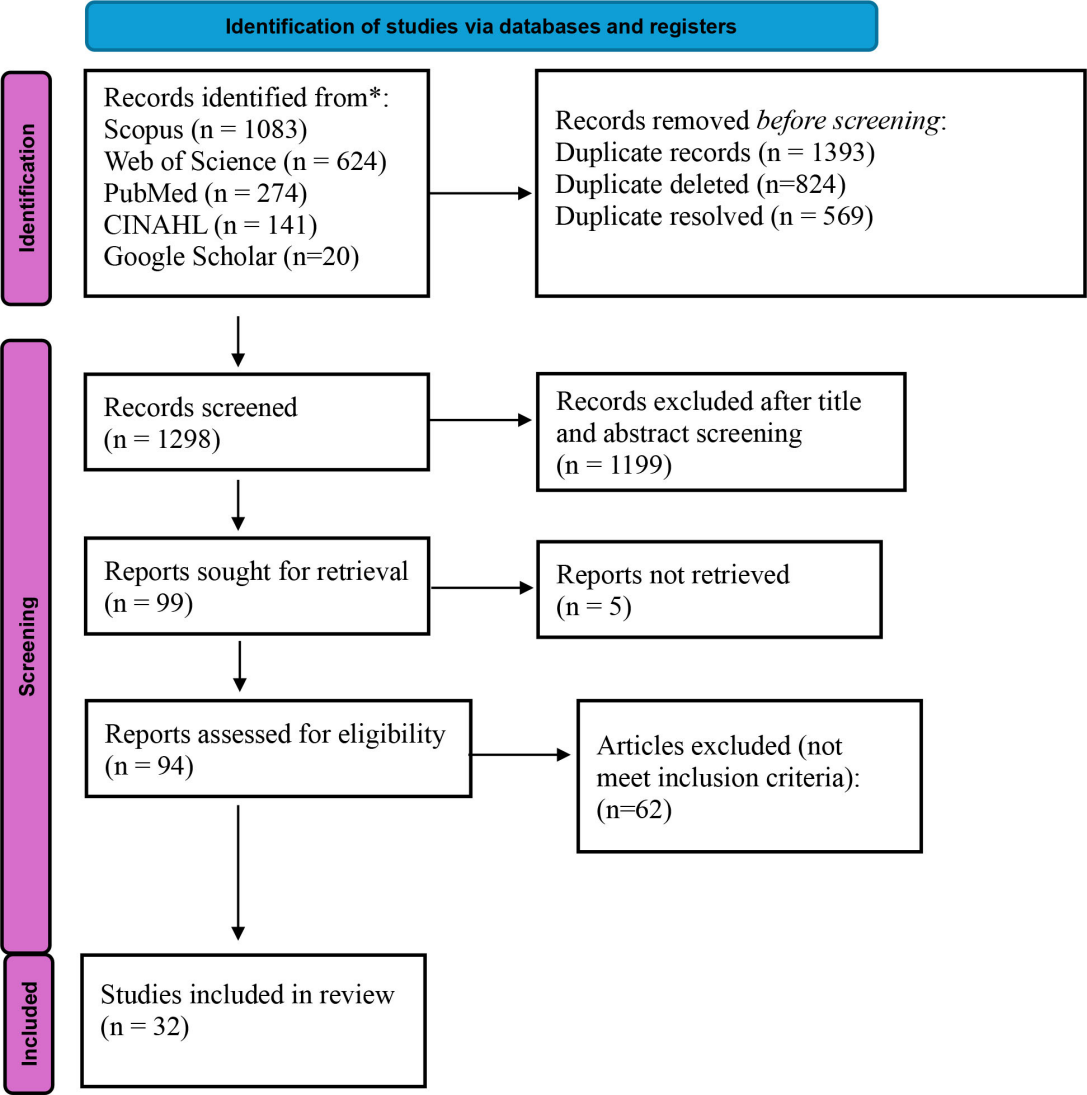
PRIMA study selection flow chart.

The systematic search identified 2,142 records across all databases (PubMed: 274, Scopus: 1083, Web of Science: 624, CINAHL: 141, Google Scholar: 20). After removal of 824 duplicates, 1,298 records underwent title and abstract screening. Of these, 1199 were excluded as irrelevant, leaving 99 full-text articles for detailed assessment. Following full-text review, 61 articles were excluded for the following reasons: non-Nigerian participants or age <18 years (n=18), did not report prevalence, risk factor associations, or management data (n=33), insufficient data for extraction (n=5), report not retrieved (n=5), and ineligible study designs (n=4). Ultimately, 32 studies met all inclusion criteria and were included in the systematic review and meta-analysis (**Figure 2**).

### 3.2 Risk of bias assessment

**Figure 3:**
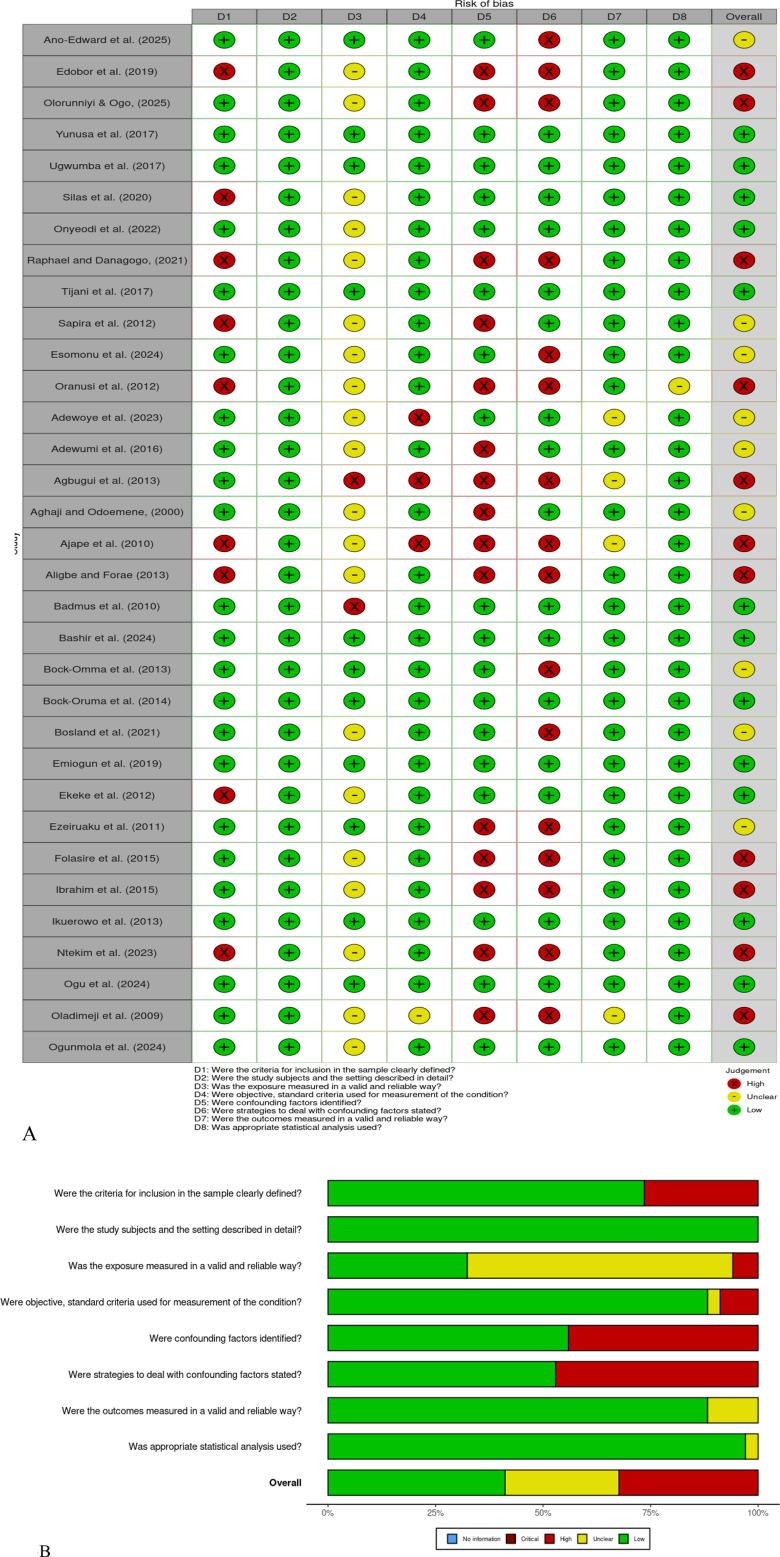
**(A)** Traffic-light plot presenting individual study-level judgments for each domain. **(B)** Summary plot of risk of bias across methodological domains.

Risk of bias assessment revealed that 13 studies (39.4%) had low risk of bias, 9 studies (27.3%) had moderate risk, and 11 studies (33.3%) had high risk of bias (**Figures 3A, B**). Common sources of bias included: inadequate description of sampling methods (n=9, 21.4%), lack of representativeness of study populations (n=2, 4.8%), insufficient adjustment for confounding in risk factor analyses (n=16, 38.1%), and cofounding factors data (n=15, 35.7%). The predominance of hospital-based studies introduced inherent selection bias, as patients attending healthcare facilities may differ systematically from the general population in terms of disease severity, health-seeking behaviour, and socioeconomic status.

### 3.3 Study characteristics

The 32 included studies comprised a total of 19,050 participants and were published between 2000 and 2025 (**Table 1**). Study designs included retrospective cohort studies (n=15, 44.1%), cross-sectional studies (n=14, 41.2%), prospective cohort studies (n=4, 11.8%), and one preliminary report (n=1, 2.9%). The majority of studies were hospital-based (n=28, 82.4%), with fewer community-based (n=3, 8.8%) or population-based studies (n=3, 8.8%).

**Table 1:** Study characteristics of the reviewed studies.

State	Setting	Study design	Period of data collection	Sample size	No. of cases	Study population	Mean age	Case definition	Reference
Oyo	Hospital	Retrospective	2017–2021	149	149	NA	72.6	Histology	Ano Edward et al. (31)
Lagos	Hospital	Retrospective	2015 – 2018	717	333	46.40%	69 ± 8	Histology	Emiogun et al. (32)
Ogun	Hospital	Cross-sectional study	2024	128	NA	NA	40–70	NA	Olorunniyi & Ogo (33)
Kano	Hospital	Cross-sectional	2012	93	35	37.6%	68.1 ± 9.4	Histology	Yunusa et al. (34)
Enugu	Community-based	Cross-sectional	2016	160	3	1.88%	55.5	PSA, DRE, Histology	Ugwumba et al. (35)
Plateau	Population-based	Retrospective cohort	2016 – 2018	930	150	16.10%	50	Histology	Silas et al. (36)
Lagos	Communities-based	Cross-sectional	2019	270	NA	NA	49.4 ± 8	PSA, DRE	Onyeodi et al. (37)
Rivers	Hospital	Retrospective	2011-2020	278	278	NA	68.39	Histology	Raphael & Danagogo (38)
Lagos	Hospital	Prospective study	2013-2015	169	37	22%	63 ± 6.5	Histology	Tijani et al. (39)
Rivers	Hospital	Retrospective study	1996-2010	383	383	NA	70.3 ± 2.5	Histology	Sapira et al. (40)
Cross river	Hospital	Cross-sectional study	2023	615	NA	0.81%	44-80	Clinical, Imaging	Esomonu et al. (41)
Anambra	Public servant	Cross-sectional	2011-2012	652	NA	NA	45.1	NA	Oranusi et al. (42)
Ekiti	Hospital	Cross-sectional	2022-2023	340	NA	NA	53.7 ± 11.4	NA	Adewoye et al. (43)
Lagos	Hospital	Retrospective	2001-2010	144	144	23.60%	66.19 ± 7.3	Histology	Adewumi et al. (44)
Edo	Urban centre	Cross-sectional study	2012	402	NA	NA	51 ± 8.2	NA	Agbugui et al. (45)
Enugu	Hospital	Prospective study	1989-1998	847	39	4.60%	71.6	Histology	Aghaji and Odoemene (46)
Kwara	Population-based	Retrospective	1997-2006	192	90	6.65%	68.4 ± 10.1	Histology, PSA, Clinical, Registry	Ajape et al. (47)
Edo	Hospital	Retrospective study	2001-2010	908	201	22.10%	43-103	Histology	Aligbe and Forae (48)
Osun	Hospital-based	Retrospective study	1991-2007	189	NA	NA	68 ± 9.8	Histology	Badmus et al. (49)
Kano	Hospital	Prospective	2014	87	25	28.70%	68.1	DRE, PSA, Imaging, Histology	Bashir et al. (50)
Rivers	Hospital	Prospective	2010-2012	290	37	12.50%	65.5 ± 11.6	DRE, PSA	Bock-Oruma et al. (51)
Rivers	Hospital	Prospective	2010-2012	290	NA	NA	62.5 ± 11.55	DRE, PSA	Bock-Oruma et al. (52)
Lagos	Hospital	Preliminary report	2017-2018	39	3	7.7%	55 ± 11	Histology, PSA, DRE	Bosland et al. (53)
Rivers	Hospital	Retrospective	2003-2012	294	216	73.47%	69.9	Histology	Ekeke et al. (54)
Rivers	Community-based	Cross-sectional study	2007-2010	1325	41/99	41.40%	40-80	Histology	Ezeiruaku et al. (55)
Oyo	Hospital	Retrospective study	2006-2010	82	82	NA	67 ± 1.8	Histology	Folasire et al. (56)
Borno	Hospital	Retrospective	2009-2013	101	101	NA	67.3	Imaging	Ibrahim et al. (57)
Lagos	Hospital	Retrospective study	2013	4110	NA	1.05%	60.8	Histology	Ikuerowo et al. (58)
National	Hospital	Retrospective	2009-2016	4864	355	8.86%	55	NA	Ntekim et al. (59)
Enugu	Hospital	Cross-sectional study	2023	66	9	15.80%	51 ± 9.6	PSA	Ogu et al. (60)
Oyo	Communities	Cross-sectional study	2009-2010	561	NA	NA	62.6 ± 9.97	NA	Oladimeji et al. (61)
Kogi	Hospital	Retrospective study	2016–2020	83	83	NA	70.4 ± 10.6	Histology	Ogunmola et al. (62)

Geographically, studies were distributed across all six geopolitical zones of Nigeria: Southwest (n=11, 32.4%), South-South (n=8, 23.5%), Southeast (n=4, 11.8%), North-Central (n=4, 11.8%), Northwest (n=4, 11.8%), and Northeast (n=2, 5.9%). One study was national in scope. The data collection periods ranged from 1989 to 2024, with sample sizes varying from 39 to 4,864 participants (median: 270). Mean age of participants ranged from 40 to 72.6 years across studies. Case definitions varied: histological confirmation was used in 24 studies (70.6%), PSA and/or DRE in 8 studies (23.5%), imaging in 3 studies (8.8%), clinical diagnosis in 2 studies (5.9%), and cancer registry data in 1 study (2.9%). Some studies employed multiple diagnostic modalities.

### 3.4 Case definitions

**Figure 4:**
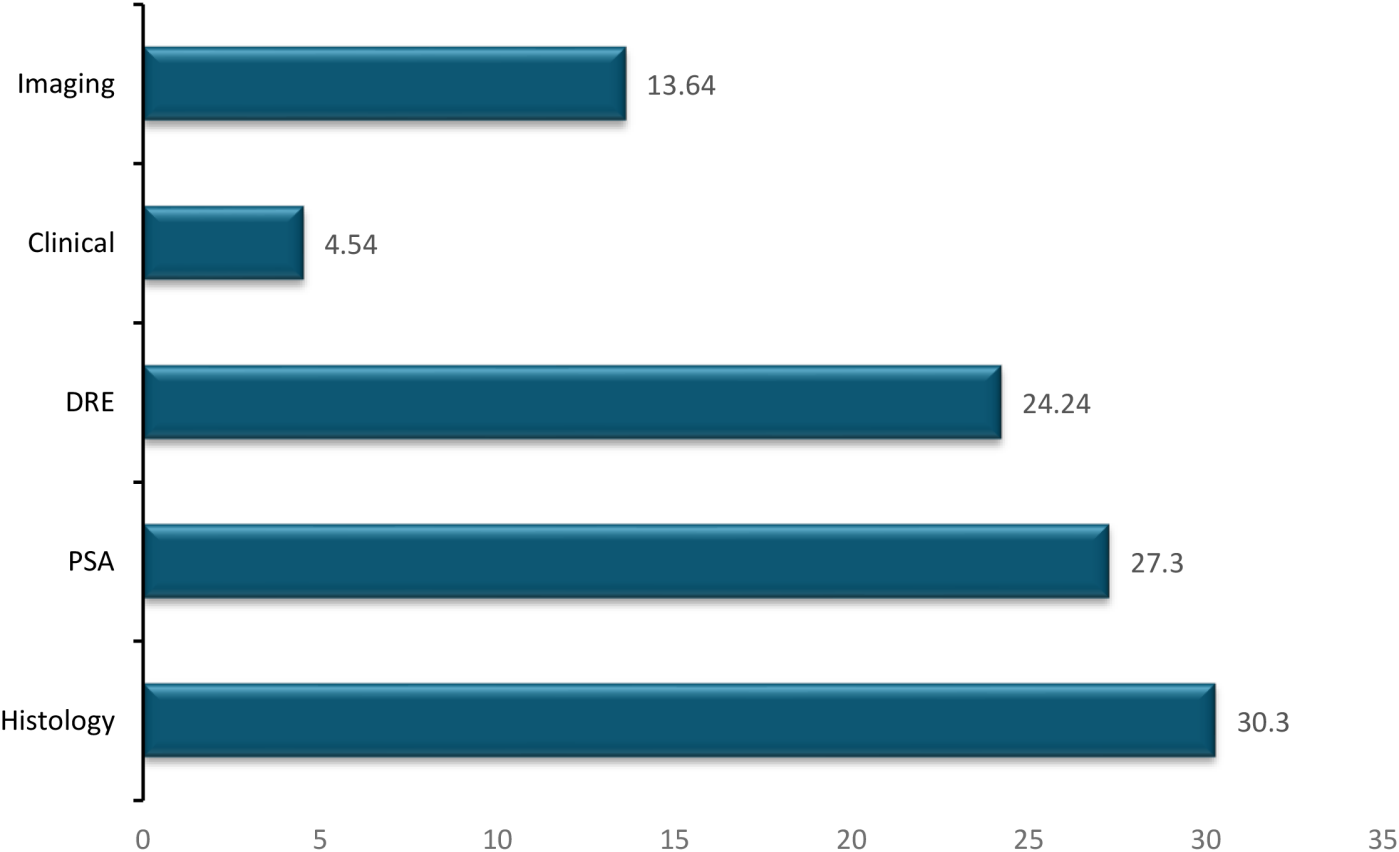
The percentage distribution of case definition mentioned in some of the Included Articles.

Among the 32 included studies, case definitions varied considerably (**Figure 4**). Histological confirmation (biopsy or surgical specimen) was the most common method (30.3% of studies), followed by PSA testing (27.3%) while digital rectal examination (DRE) (24.24.3%), imaging modalities such as ultrasound or MRI (13.64%), clinical diagnosis based on symptoms and physical examination (5.54%). Several studies employed multiple diagnostic methods. The variability in case definitions contributes to heterogeneity in reported proportions and limits comparability across studies.

### 3.5 Meta-analysis

#### 3.5.1 Hospital-based proportion of prostate cancer

**Figure 5:**
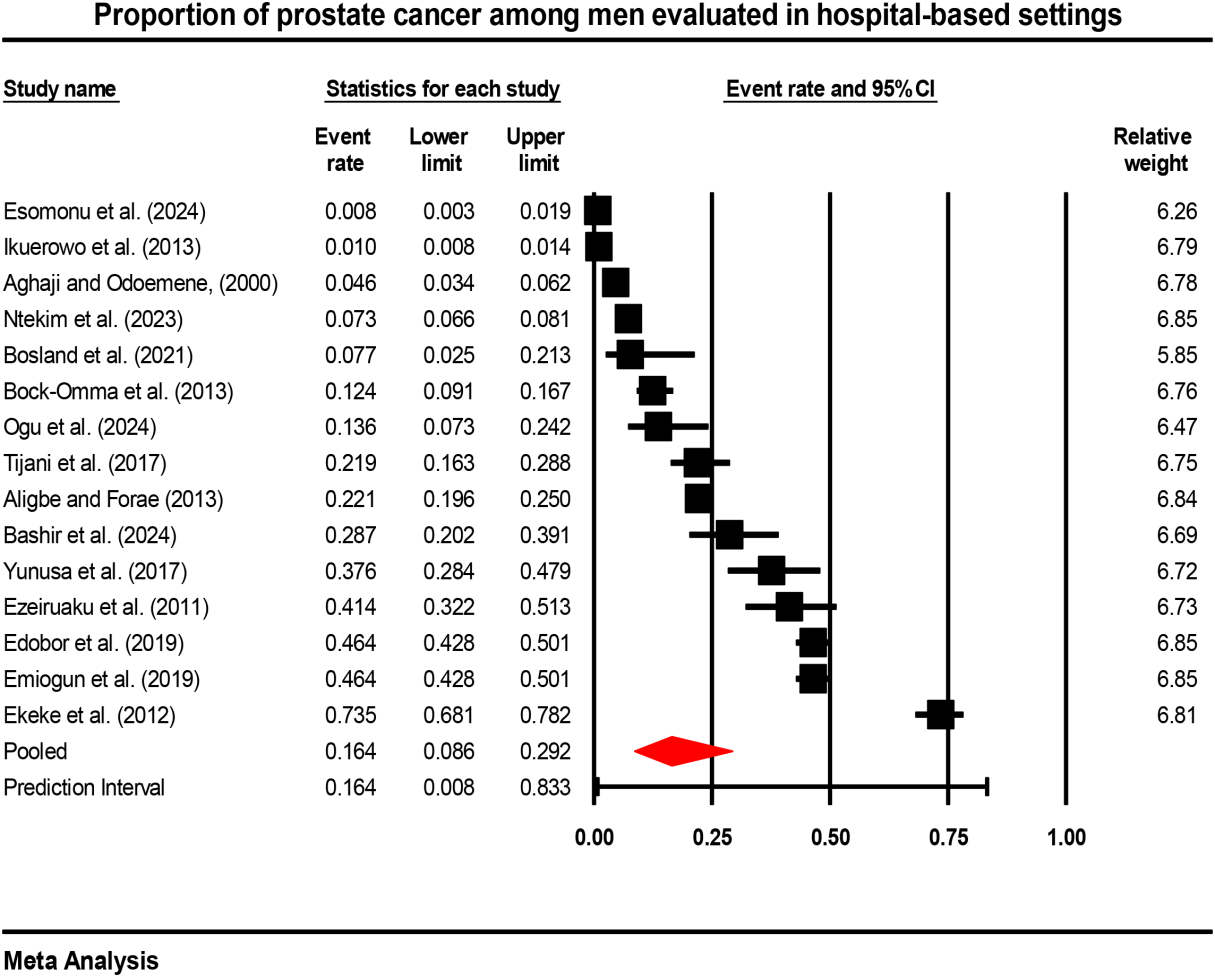
Forest plot showing hospital-based proportion of prostate cancer in Nigeria (random-effects model).

Meta-analysis of 15 hospital-based studies yielded a pooled proportion of prostate cancer of 16.4% (95% CI: 8.6%–29.2%) (**Figure 5**). The 95% prediction interval ranged from 0.8% to 83.3%, indicating substantial variability in the proportion of prostate cancer across different hospital settings. Heterogeneity was extreme, with I²=99.3%, Cochran’s Q = 1970.5 (df=14, p<0.001), τ²=2.093, and τ=1.447. The very high I² statistic indicates that 99.3% of the total variation in proportion estimates is due to between-study heterogeneity rather than sampling error. This 16.4% represents the proportion of prostate cancer among patients attending hospital-based urology, oncology, or surgical services—not the prevalence in the general Nigerian population. These are facility-based proportions reflecting the case mix in specialized clinical settings and cannot be generalized to estimate national or population-level prevalence.

#### 3.5.2 Population-based proportion of prostate cancer

**Figure 6:**
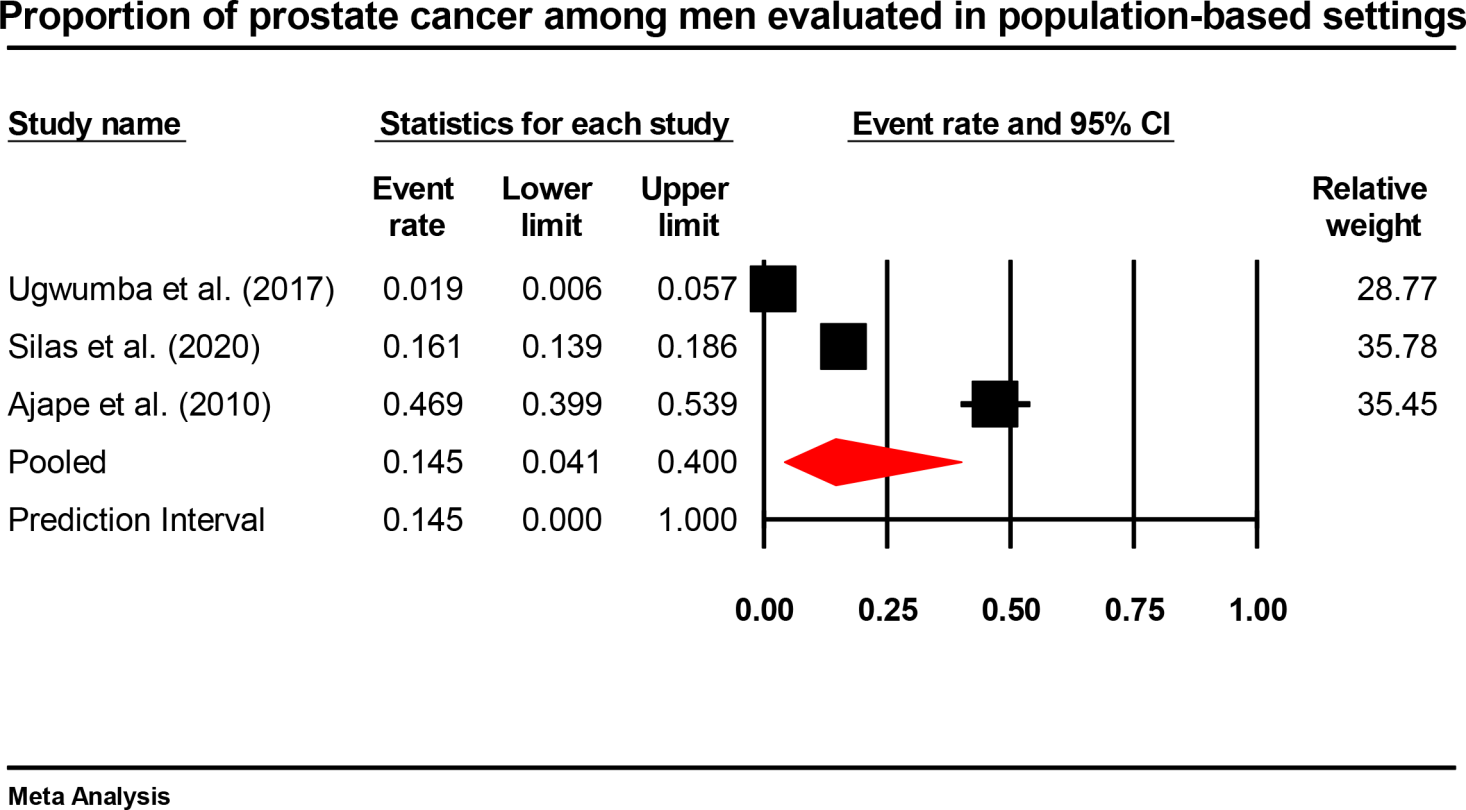
Forest plot showing population-based proportion of prostate cancer in Nigeria (random-effects model).

A separate analysis of three population-based or community-based studies yielded a pooled proportion of 14.0% (95% CI: 4.1%–40.0%; prediction interval: 0.5%–84.6%) with similarly extreme heterogeneity (I²=98.0%, Q = 101.964, df=2, p<0.001, τ²=1.352, τ=1.163) (**Figure 6**). These estimates also reflect screening or case-finding activities in selected communities rather than true population prevalence, as they were not derived from representative population-based cancer registries.

#### 3.5.3 Subgroup analyses

Subgroup analyses revealed substantial variation in hospital-based proportions across multiple study characteristics (**Table 2**): Proportions varied significantly by region (p=0.563 for between-subgroup difference, though not statistically significant due to small numbers): Northwest (33.0%, 95% CI: 6%–79%), North-Central (29.1%, 95% CI: 5%–76%), South-South (19.5%, 95% CI: 6%–47%), Southwest (15.5%, 95% CI: 4%–41%), Northeast (7.0%, 95% CI: 0.4%–58%), and Southeast (5.1%, 95% CI: 1%–23%). These regional differences likely reflect variations in healthcare access, screening practices, referral patterns, and diagnostic capacity rather than true differences in disease burden. Tertiary care centres reported significantly higher proportions (27.0%, 95% CI: 14%–46%) compared to primary care settings (9.1%, 95% CI: 4%–19%) (p=0.033). This difference is expected given that tertiary centres serve as referral hubs for complex cases and cancer care. Single-centre studies reported higher proportions (25.0%, 95% CI: 13%–42%) than multi-centre studies (7.4%, 95% CI: 3%–18%) (p=0.027), possibly reflecting differences in case mix and referral patterns at individual institutions. Studies with mean participant age >60 years reported higher proportions (22.5%, 95% CI: 14%–35%) compared to those with mean age ≤60 years (8.0%, 95% CI: 3.5%–17%) (p=0.029), consistent with the known age-related increase in prostate cancer incidence. No significant differences were observed between studies with sample sizes above versus below 100 participants (p=0.373) or between earlier (1995–2015) versus more recent (2016–2026) data collection periods (p=0.323).

**Table 2:** Subgroup and meta-regression analyses of potential sources of heterogeneity.

Variables	N	Proportion	Prediction interval	df (Q statistic)	τ	τ^2^	P-value	Intercept	R^2^ analog
Setting									
Population based	3	14.0 (0.03 – 0.45)	0.005 – 0.846	1 (0.041)	1.428	2.039	0.839	**-**1.44 (-4.19 – 1.31)	0.00
Hospital-based	15	16.4 (0.086 – 0.29)	0.009 – 0.818						
Overall	18	16.0 (0.089 – 0.27)	0.010 – 0.786						
Zone									
Northcentral	2	29.1 (0.05 – 0.76)	0.009 – 0.95	5 (3.910)	1.470	1.470	0.563	-1.92 (-4.29 – 0.44)	0.04
Northeast	1	7.0 (0.004 – 0.58)	0.004 – 0.58						
Northwest	2	33.0 (0.06 – 0.79)	0.059 – 0.795						
Southeast	3	5.1 (0.01 – 0.23)	0.010 – 0.232						
Southsouth	5	19.5 (0.06 – 0.47)	0.008 – 0.881						
Southwest	5	15.5 (0.04 – 0.41)	0.006 – 0.850						
Level of Care									
Primary	9	9.1 (0.04 – 0.189)	0.006 – 0.63	1 (4.571)	1.262	1.594	**0.033**	-3.067 (-3.23 - -2.90)	0.13
Tertiary	9	27.0 (0.14 – 0.462)	0.010 – 0.785						
Number of Centre									
Multi-centre	7	7.4 (0.029 – 0.177)	0.004 – 0.610	1 (4.872)	1.308	1.711	**0.027**	0.326 (1.06 - 1.92)	0.07
Single Centre	11	25.0 (0.132 – 0.42)	0.018 – 0.858						
Sample Size									
Below Hundred	6	13.8 (0.07 – 0.252)	0.008 – 0.763	1 (0.793)	1.363	1.857	0.373	-0.519 (-3.81 – 2.77)	0.00
Above Hundred	12	23.6 (0.083 – 0.51)	0.013 – 0.881						
Mean Age of Respondent									
≤ 60 Years	6	8.0 (0.035 – 0.17)	0.007 – 0.506	1 (4.77)	1.069	1.143	**0.029**	-0.03 (-2.35 – 2.28)	0.38
> 60 Years	12	22.5 (0.14 – 0.35)	0.027 – 0.756						
Years of data collection									
1995 – 2015	11	19.6 (0.10 – 0.348)	0.007 – 0.753	1 (0.978)	1.312	1.722	0.323	-0.764 (-3.22 - 1.69)	0.06
2016 – 2026	7	11.4 (0.04 – 0.261)	0.012 – 0.821						

Q, Cochrane Q; τ, tau, τ^2,^ tau square. Bold values indicate statistically significant results (p < 0.05)

#### 3.5.4 Meta-regression analysis

Random-effects meta-regression examined the association between study-level covariates and log odds of prostate cancer proportion (**Table 2**). Neither mean age of participants (coefficient=0.104, 95% CI: -0.049 to 0.256, p=0.171) nor year of data collection (coefficient=0.004, 95% CI: -0.169 to 0.176, p=0.966) showed significant associations with prostate cancer proportion. The combined model including both covariates explained only 22% of between-study variance (R²=0.22), indicating that the majority of heterogeneity remains unexplained by these factors. Between-study variance remained high (τ²=1.857, I²=99.1%), suggesting that other unmeasured factors—such as differences in diagnostic criteria, patient selection, healthcare infrastructure, and referral patterns—contribute substantially to heterogeneity.

#### 3.5.5 Sensitivity analysis and publication bias

**Figure 7:**
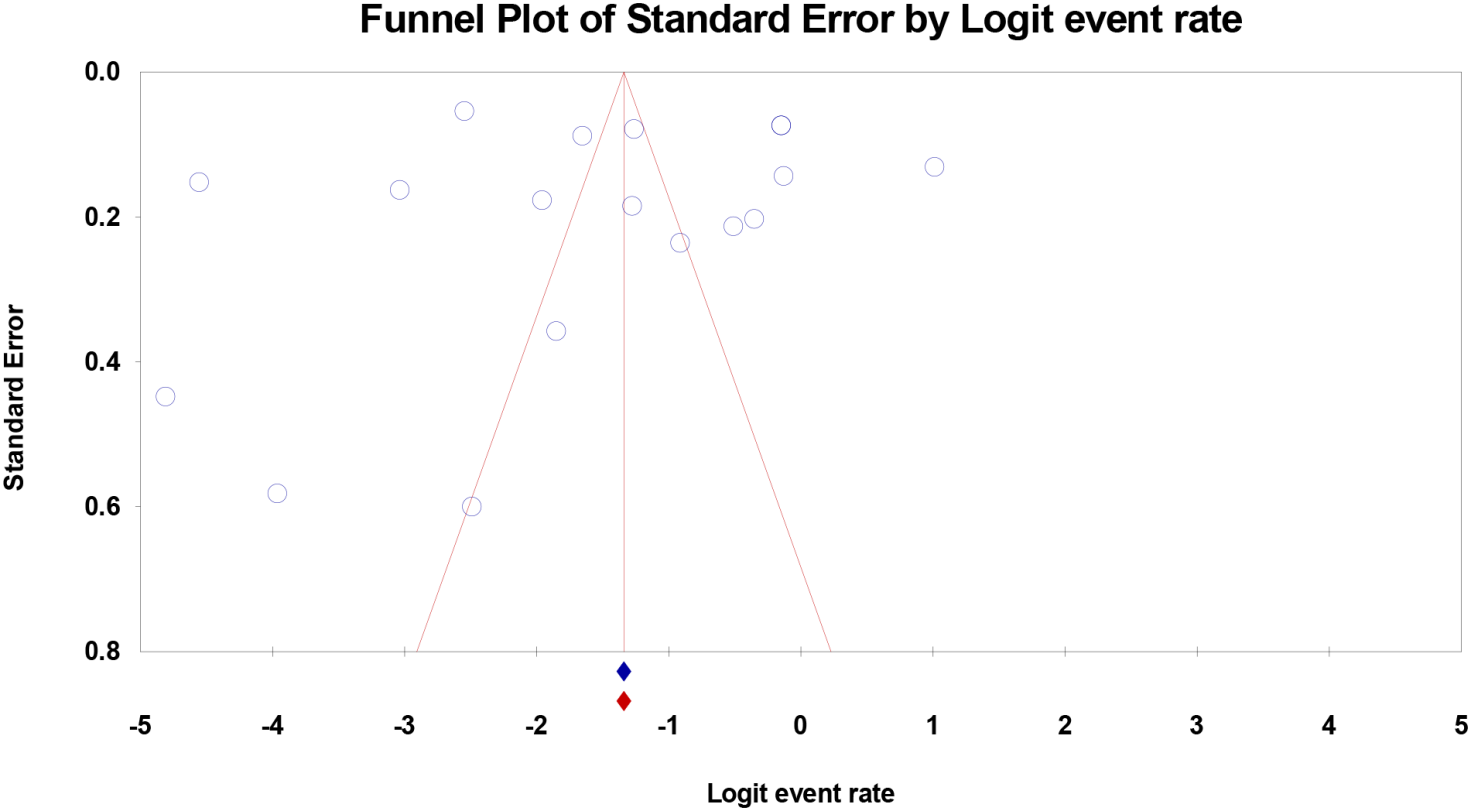
Funnel plot for studies on prostate cancer prevalence in Nigeria. Open diamond indicates point estimates from observed studies while the closed diamond indicates point estimates from imputed studies.

The sensitivity analysis of leave-one-out showed that none of the studies had a disproportional influence on the pooled estimate; exclusion of any of the studies led to pooled proportions between 15.1 and 17.8, indicating the strength of the entire result. The visual inspection of the funnel plot indicated that it was asymmetrical which likely meant a publication bias. The regression test by Egger was statistically significant (p=0.041), which implies small-studies effects. Trim-and-fill analysis was used to suspiciously impute 5 possibly missing studies on the left side of the funnel and after correction the pooled proportion became 13.2% (95% CI: 7.1%-23.4%), which is slightly less than the unadjusted estimate but still implies a large proportion of hospitals (**Figure 7**).

### 3.6 Risk factor proportions among cases

**Figure 8:**
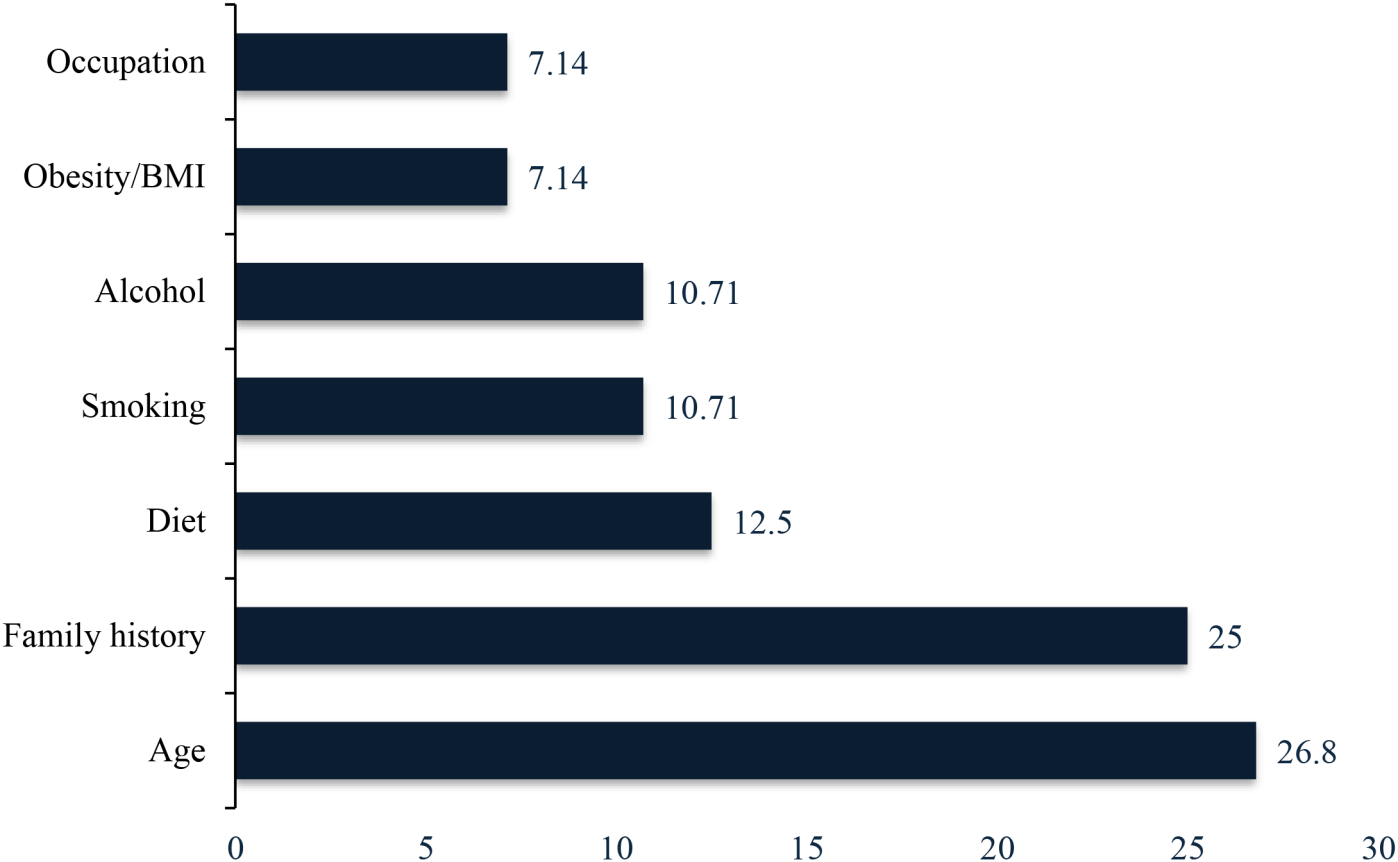
The percentage distribution of risk factors mentioned in some of the Included Articles.

Among the prostate cancer cases that were discovered in all the studies included, 26.8% of prostate cancer cases were identified to be caused by old age, 25% by family history, 12.5% by diet, 10.7% by smoking, 10.7% by alcohol use, 7.14% by obesity (BMI ≥ 30 kg/m²), and 7.1% by occupation (**Figure 8**). Such values are descriptive frequencies, which indicate a proportion of cases, having each characteristic. Nor do they reflect the risk associations, odds ratios, and relative risks, as most of the studies involved in the studies did not have proper comparison groups and did not control the risk of potential confounders. Thus, such results cannot prove causal relationships and must be regarded as observed patterns between cases, but not be seen as evidence of the independent effects of risk factors.

### 3.7 Treatment modalities

**Figure 9:**
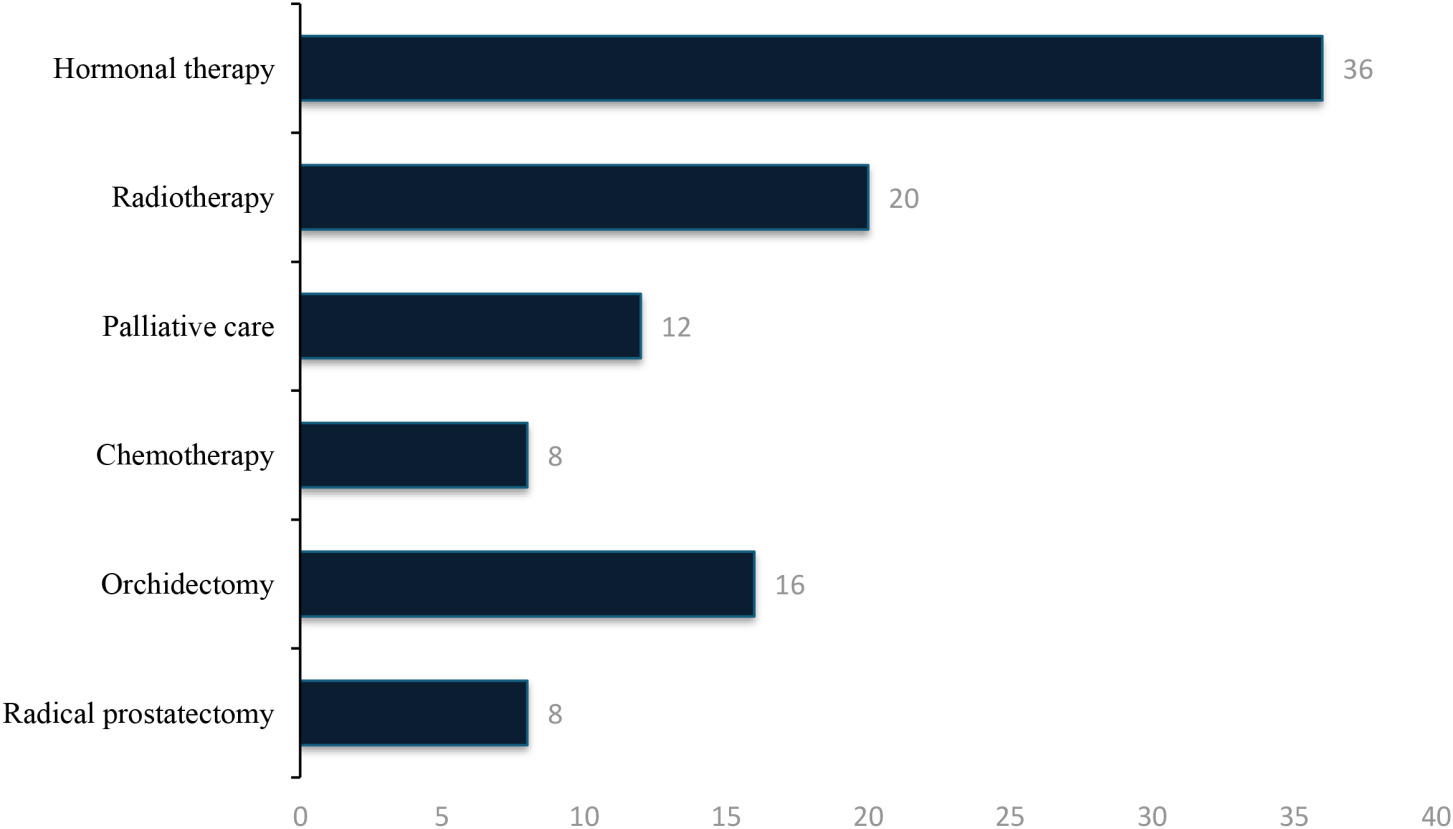
The percentage distribution of treatment modalities mentioned in some of the Included.

Treatment patterns among prostate cancer patients are presented in **Figure 9**. Many patients received multiple therapeutic modalities, either sequentially or in combination, which accounts for total treatment proportions of 100%. Hormonal therapy (ADT) was administered to 36% of patients, including surgical castration (orchiectomy) or medical castration with GnRH agonists or antagonists. External beam radiotherapy was received by 20% of patients, Palliative care 12%, systemic chemotherapy by 8%, radical prostatectomy by 8%, and Orchidectomy in 16% of cases. The predominance of hormonal therapy (ADT) likely reflects its wider availability and relative affordability compared with other treatment modalities. The very low rate of radical prostatectomy suggests limited access to surgical expertise and infrastructure for curative treatment, while restricted radiotherapy utilization aligns with the limited number of radiotherapy centres in Nigeria.

### 3.8 GRADE certainty of evidence

The certainty of evidence was rated low for all outcomes (**Table 3**) due to risk of bias in most studies, extreme heterogeneity, indirectness from hospital-based data, imprecision from wide confidence intervals, and evidence of publication bias. Consequently, the true effects may differ substantially from the reported estimates, and the findings should be interpreted with limited confidence.

**Table 3:** GRADE certainty of evidence assessment.

Outcome	Hospital-based proportion	Population-based proportion
Study Design	Observational	Observational
Risk of Bias	Serious (–1)	Serious (–1)
Inconsistency	Serious (–1)	Serious (–1)
Indirectness	Not serious (0)	Not serious (–0)
Imprecision	Serious (–1)	Serious (–1)
Publication Bias	Serious (–1)	Serious (–1)
**Certainty**	**Low⊕⊕◯◯**	**Low⊕⊕◯◯**

Bold value indicated key summary estimated certainty of the result outcome which is low.

## 4 Discussion

This systematic review and meta-analysis offer an in-depth synthesis of the burden and clinical features and management trends of prostate cancer in Nigeria within 25 years (2000–2025). We have found that among the hospital-based diagnoses, prostate cancer is the largest and that the pooled estimate of 16.4 (95% CI: 8.6%-29.2) is among men who have visited the healthcare facilities. This number is similar to those of other sub-Saharan African nations where the proportions in the hospitals are between 14-22% (3). Nevertheless, the high level of heterogeneity (I^2^ = 99.3) indicates the high level of heterogeneity in case ascertainment, healthcare accessibility and diagnostic capacity across the institutions in Nigeria.

The hospital-wide rate of 16.4% means that one out of six men who attend a healthcare center in Nigeria with urological or oncological problems is diagnosed with prostate cancer. This observation is consistent with the recent multicentric research studies conducted in West Africa with prostate cancer percentages of 14–18 percent of patients with symptoms (12, 63). Nevertheless, it is still problematic to make direct comparisons with population-based incidence rates in high-income countries as the estimates we have are facility-based case mix, not actual population prevalence. In 2020, the Nigerian National System of Cancer Registries estimated age-standardized incidence rates at 15.6 per 100, 000 (64) but these figures do not provide a true picture of the burden because of incomplete case reporting and overall coverage (1).

It is important to note that the three population-based studies that we included in our analysis gave an identical pooled proportion of 14.0% (95% CI: 4.1%-40.0%), indicating that the population-based screening activities yield similar rates of cases. The studies however used opportunistic sampling as opposed to rigorous epidemiological surveys and thus could not be generalized. The large range of prediction values of both hospital-based (0.8%-83.3%) and population-based (0.5%-84.6) indicate the high level of variation within the Nigerian settings and dissuades the use of the pooled estimates in national policy planning without references to specific settings (65).

It was found that there were very large regional discrepancies where the proportions were higher in Northwest (33.0%), and North-Central (29.1%), than the proportions were in the Southeast (5.1%), and Northeast (7.0%). Although the differences were not statistical significance with the lack of studies per region, they indicate that there are significant geographic variations in the detection of diseases and the infrastructure of healthcare. Recent mapping of oncology services in Nigeria reported that 68 percent of the radiotherapy facilities and 75 percent of urology oncology experts are all found in the Southwest and South-South regions (58, 59). Ironically, higher percentages were found in more healthcare concentrated areas which may indicate higher rates of benign prostatic hyperplasia diagnosis or more stringent diagnostic algorithms which prevent false positives. The increased percentages in the northern areas could be a result of late presentation and later disease on diagnosis, which is in line with the literature that demonstrates that patients in northern Nigeria cover more distance to access care and present with a higher level of PSA (66). Recent multicenter cohort study has shown that the median PSA at diagnosis was 98 ng/mL in Kano and 42 ng/mL in Lagos with the relevant differences in the rates of metastatic disease (67). Such geographic differences explain why it is important to have decentralized cancer services and specific screening initiatives in underserved areas (68).

The significantly higher proportions at tertiary care centres (27.0%) compared to primary care settings (9.1%) (p=0.033) is consistent with the role of tertiary centres as referral hubs for complex cases and cancer care (49). This finding underscores the importance of stratifying analyses by level of care when interpreting facility-based data (69). The higher proportions in single-centre studies (25.0%) compared to multi-centre studies (7.4%) (p=0.027) may reflect differences in case mix, with single-centre studies often conducted at specialized cancer centres with high concentrations of cancer patients (42). Multi-centre studies, by including a broader range of facilities, may provide more representative estimates of the overall healthcare system burden (48). Leave-one-out sensitivity analysis confirmed the robustness of the pooled estimate, with no single study exerting disproportionate influence. However, evidence of publication bias (funnel plot asymmetry, significant Egger’s test) suggests that small studies with lower proportions may be underrepresented in the published literature (70). Trim-and-fill analysis adjusted the pooled proportion downward to 13.2%, though this remains a substantial facility-based proportion.

Histological confirmation was employed in 70.6% of studies, representing a strength of the Nigerian literature compared to some other African settings where clinical diagnosis predominates (71). However, the variability in diagnostic pathways—with 27.3% of studies relying solely on PSA and 13.6% on imaging without biopsy—introduces substantial heterogeneity and potential misclassification. Recent guidelines from the Nigerian Institute for Cancer Research and Treatment emphasize that PSA screening without histological confirmation is inadequate for definitive diagnosis, particularly in populations with high rates of prostatitis and benign prostatic hyperplasia (72).

Average age of prostate cancer cases (51 to 72.6 years) is significantly low compared to the West populations where the median age is diagnosed at more than 66 (73). This result corresponds to the African-Caribbean cancer disparity hypothesis, which states that, men of African origin are more likely to develop prostate cancer at a younger age and have more aggressive cancer (74). A genomic analysis on prostate tumors of Nigerian men detected unique somatic changes such as an increased proportion of ERG fusions and loss of PTEN, both of which could be the reason behind earlier onset and aggressive phenotype (75). Such differences in the biology emphasize the need to come up with population-specific risk stratification instruments and screening guidelines of Nigerian men (5).

The descriptive statistics of risk factors among cases older age (26.8%), family history (25%), dietary factors (12.5%), smoking (10.7%), and alcohol use (10.7%) give an idea of the nature of afflicted men but are not a causal study. The reason why the high proportion is in line with recent African consortia studies which have shown that family history accounts to 15–25 percent of prostate cancer incidence in sub-Saharan Africa as opposed to 10–15 percent in the European populations (76, 77). The patterns of genetic vulnerability to TB have shown to be unique as genome-wide association studies in Nigerian cohorts have been able to identify novel risk loci that were not detected in other ancestry populations (78, 79). The comparatively low reported prevalence of modifiable risk factors in the cases may indicate under-ascertainment instead of lack of association related to the risks. According to a recent case-control study conducted in Ibadan, there was a significant red meat consumption (OR: 2.4, 95% CI: 1.3 4.2) and physical inactivity (OR: 1.9, 95% CI: 1.1 3.1) with prostate cancer, after the researchers adjusted the results based on confounding factors (80). Yet, the largest number of studies did not have relevant comparison groups and the control of confounding, which restricts causation inference. Futuristic research ought to use rigorous designs of analysis with population based controls to measure associations of risk factors (81).

Patterns of treatment analysis indicate that the most common therapy includes hormonal therapy (36%), and a much less common usage of curative-intent therapy: radiotherapy (20%), radical prostatectomy (8%), and systemic chemotherapy (8%). Those trends are indicative of deep inequalities in cancer care access relative to high-income nations, in which surgery and radiotherapy are first-line cancer treatment in more than 70% of cases of localized disease (82). It was recognized in the Nigerian National Cancer Control Plan 20182022 that there were only three operating radiotherapy centers serving more than 200 million people one of the lowest radiotherapy densities in the world (64). This 16 percent orchidectomy (surgical castration) is comparatively high in comparison to current Western practice (GnRH agonist). This difference is based on economic reality: orchidectomy costs about 50,000-100,000 (USD120-240) as compared to annual GnRH agonist expenditures of over 500,000 (USD1200) that is prohibitive to most Nigerians since 40 percent live below poverty line (83). An analysis of cost-effectiveness of orchidectomy in Nigeria found that orchidectomy is the cheapest form of androgen deprivation technique but has a high psychological morbidity (84).

Low radical prostatectomy rate (8 per cent) is a cause of concern especially considering the fact that men with localized disease may have curative surgeries. Obstacles are few urology oncologists (estimated less than 20 in the country), lack of robotic surgical systems, and expensive out-of-pocket expenses (₦1.5–3 million, US$3,6007200) (85, 86). Likewise, the use of radiotherapy is limited by the geographic coverage of radiotherapy facilities-patients in the northeast part of the country have to travel a distance of more than 1,000km to access the nearest operating radiotherapy facility in Lagos or Zaria, which create high indirect costs and delays in the treatment process (87).

The vast prevalence of hospital-based researches (82.4%), and single-centre designs (61.8%), demonstrates the fragmented research and care of prostate cancer in Nigeria. In comparison to South Africa that has developed national cancer registries and multicentre research networks, Nigeria does not have a coordinated infrastructure of data collection (88). The National Health Insurance Scheme does not cover over a fifth of the population and offers minimal benefits oncology coverage meaning that most patients have to self-finance their care expenses via out-of-pocket payments (89). Recent survey of prostate cancer patients in Lagos indicated that, 78 percent had catastrophic health outlay, 34 percent discontinued treatment because of economic limitations (90).

This 16.4% pooled hospital-based proportion in Nigeria is comparable with that found in Ghana (18.2%), Cameroon (15.7%), and Senegal (14.9) in recent systematic reviews (9). Nevertheless, these estimates are considerably greater than hospital-based rates in East Africa (Tanzania: 8.3%, Uganda: 9.1%), indicating that there is a regional difference in disease burden or healthcare access and usage rates (46). Globally, the incidence of prostate cancer is > 60-fold with the highest rates among Caribbean men of African origin (age-standardized rate: 158.3 per 100,000 in Barbados) and the lowest among Asian populations (91). The Nigerian incidence estimates (15.6 per 100,000) probably demonstrate tremendous underreporting as a result of unfinished case ascertaining, and modelling research hypothesizes that the actual rates would be 50–70 per 100,000 (92).

Policy and Practice implication Our study has a number of implications in cancer control in Nigeria. To begin with, the overwhelming heterogeneity and hospital-based data highlights the pressing importance of the population-based cancer registries with full ascertainment of cases and universal diagnostic criteria. The increased number of the Nigerian National System of Cancer Registries to 12 population-based registries is progress, yet more funding and technical assistance is needed (93). Second, the geographic disparities in the reported proportions demonstrate the necessity of decentralized cancer services, such as an increase in the radiotherapy capacity to every geopolitical region and the shifting of prostate biopsy to trained general surgeons in low-density regions (94).

Third, the prevalence of hormonal treatment and low curative treatment make new funding mechanisms. The access could be enhanced by increasing health insurance coverage of cancer care, developing collaboration between the state and the market to buy equipment, and securing volume discounts on necessary cancer drugs (95). Fourth, Nigeria has a younger age of diagnosis than Western populations indicating that screening guidelines might not be applicable in low-income countries (96). The Nigerian Urological Association ought to look at the possibility of coming up with age-specific screening guidelines on the basis of the local epidemiological data.

### 4.1 Strengths and limitations

This review has a number of strengths in terms of methodology. To begin with, we were making the most of PRISMA 2020 and registered the protocol beforehand in PROSPERO which promoted transparency and minimized bias in reporting. Second, it led to the maximum search identification due to our extensive search strategy in various databases without language limitations. Third, we used methodological sound risk of bias evaluation instruments and extensive subgroup and sensitivity analysis to investigate heterogeneity. Fourth, the study has included studies that date back to 25 years (2000–2025) and this gives a historical view of the changing practices in diagnostic and management. Fifth, the treatment pattern analysis of 32 studies is the most extensive synthesis of treatment patterns of prostate cancer in Nigeria.

Several limitations warrant consideration. First, the predominance of hospital-based studies (82.4%) introduces selection bias, as facility-based proportions cannot be generalized to estimate population prevalence. Patients attending tertiary hospitals likely differ systematically from the general population in terms of disease severity, socioeconomic status, and health-seeking behavior. Second, extreme heterogeneity (I² > 98% for all pooled estimates) limits confidence in summary estimates and suggests that pooling may obscure important context-specific variations. While we conducted extensive subgroup analyses, most heterogeneity remained unexplained.

## 5 Conclusion

This systematic review and meta-analysis demonstrate that prostate cancer constitutes a substantial burden among men attending Nigerian healthcare facilities, with a pooled hospital-based proportion of 16.4% (95% CI: 8.6%–29.2%). However, extreme heterogeneity and methodological limitations across studies preclude definitive conclusions about true population prevalence. Regional disparities in reported proportions likely reflect variations in healthcare infrastructure, diagnostic capacity, and referral patterns rather than true differences in disease burden. Clinical characteristics reveal that Nigerian men present at younger ages than Western populations, supporting the need for population-specific screening guidelines. Management patterns demonstrate predominant reliance on hormonal therapy, with limited access to curative-intent treatments including radical prostatectomy and radiotherapy. These findings reflect profound health system challenges, including inadequate oncology infrastructure, geographic maldistribution of services, financial barriers to care, and limited health insurance coverage. The 16% rate of orchidectomy highlights the economic realities driving treatment decisions in resource-constrained settings.

## Data Availability

The original contributions presented in the study are included in the article/**Supplementary Material**. Further inquiries can be directed to the corresponding author.
