# Supplementary material for: Burden, clinical characteristics, and management patterns of prostate cancer in Nigeria: a systematic review and meta-analysis
Source: Front Oncol. 2026 Mar 30;16:1780152. doi: 10.3389/fonc.2026.1780152 (PMC13070789; doi:10.3389/fonc.2026.1780152)
Supplement: Supplementary file 1 [file Table1.docx]

**SCOPUS**

(TITLE-ABS-KEY("prostate cancer" OR "prostatic neoplasm*" OR "prostate carcinoma"))

AND (TITLE-ABS-KEY(Nigeria OR "South West Nigeria" OR "South-South Nigeria" OR "North West Nigeria" OR "South East Nigeria" OR "North Central Nigeria" OR "North East Nigeria")) AND (TITLE-ABS-KEY(prevalence OR proportion OR incidence OR epidemiology OR "disease burden")) AND (TITLE-ABS-KEY("risk factor*" OR determinant* OR predictor* OR "family history" OR diet* OR age)) AND (TITLE-ABS-KEY(treatment OR therap* OR management OR "hormonal therapy" OR orchidectomy OR prostatectomy OR radiotherapy OR chemotherapy OR "palliative care"))

**CINAHL**

(MH "Prostatic Neoplasms+" OR "prostate cancer" OR "prostatic neoplasm*") AND (MH "Nigeria" OR Nigeria) AND (prevalence OR proportion OR incidence OR epidemiology)

AND (MH "Risk Factors+" OR "risk factor*" OR determinant* OR predictor*) AND

(MH "Therapeutics+" OR treatment OR management OR therapy OR "hormonal therapy" OR radiotherapy OR chemotherapy OR orchidectomy OR prostatectomy OR "palliative care")

**Web of Science (Core Collection)**

TS=("prostate cancer" OR "prostatic neoplasm*" OR "prostate carcinoma") AND TS=(Nigeria OR "South West Nigeria" OR "South-South Nigeria" OR "North West Nigeria" OR "South East Nigeria" OR "North Central Nigeria" OR "North East Nigeria") AND TS=(prevalence OR proportion OR incidence OR epidemiology OR "disease burden") AND TS=("risk factor*" OR determinant* OR predictor*) AND TS=(treatment OR therapy OR management OR "hormonal therapy" OR prostatectomy OR radiotherapy OR chemotherapy

OR orchidectomy OR "palliative care")

**PubMed**

("Prostatic Neoplasms"[Mesh] OR "prostate cancer"[tiab] OR "prostatic neoplasm*"[tiab]) AND ("Nigeria"[Mesh] OR Nigeria[tiab]) AND (prevalence[tiab] OR proportion[tiab] OR incidence[tiab] OR epidemiology[Subheading]) AND ("Risk Factors"[Mesh] OR "risk factor*"[tiab] OR determinant*[tiab] OR predictor*[tiab]) AND ("Therapeutics"[Mesh] OR treatment[tiab] OR therapy[tiab] OR management[tiab] OR "Hormone Therapy"[Mesh] OR prostatectomy[tiab] OR radiotherapy[tiab] OR chemotherapy[tiab] OR orchidectomy[tiab] OR "palliative care"[Mesh])
